# The efficacy and safety of intravitreal injection of Ranibizumab as pre-treatment for vitrectomy in proliferative diabetic retinopathy with vitreous hemorrhage

**DOI:** 10.1186/s12886-022-02303-3

**Published:** 2022-02-10

**Authors:** Shengguo Li, Yan Yang, Jingling Zou, Jun Zeng, Chun Ding

**Affiliations:** Department of Ophthalmology of The Second Xiangya Hospital, Central South University, 139 Renmin Middle Road, 410011 Changsha, China

**Keywords:** Proliferative diabetic retinopathy, Intravitreal injection of Ranibizumab, VEGF, CTGF, Tractional retinal detachment

## Abstract

**Background:**

Intravitreal injection of anti-vascular endothelial growth factor (VEGF) has become first line therapy for diabetic macular edema. This study evaluated the efficacy and safety of intravitreal injection of Ranibizumab (IVR) as pre-treatment for pars plana vitrectomy in proliferative diabetic retinopathy (PDR) patients with vitreous hemorrhage.

**Methods:**

This pilot randomized controlled trial included 48 eyes with vitreous hemorrhage resulting from active PDR. Eyes were treated with IVR 1 or 3 days before vitrectomy or a sham subconjunctival injection 3 days before surgery. The occurrence of new tractional retinal detachment (TRD), total operation time, and intraoperative findings were compared. The concentrations of VEGF and connective tissue growth factor (CTGF) in aqueous humor and plasma collected at the time of IVR and vitrectomy were determined by ELISA.

**Results:**

None of the patients who received IVR experienced new TRD. Ranibizumab injection improved intraoperative outcomes. The mean concentrations of VEGF in aqueous humor were significantly lower after than before IVR in patients who received IVR 1 and 3 days before surgery (*P* < 0.001 each). The CTGF/log_10_ (VEGF) ratio was significantly higher after than before IVR in patients who received IVR 3 days before vitrectomy (*P* = 0.046).

**Conclusion:**

Preoperative IVR is an effective and safe strategy for the surgical treatment of severe PDR combined with vitreous hemorrhage. IVR 1 and 3 days before surgery can significantly reduce VEGF content in aqueous humor and effectively improve intraoperative conditions without causing TRD.

**Trial registration:**

This study was registered with the Chinese Clinical Trial Registry. Name of the registry: Exploratory analysis of effect of intravitreal ranibizumab as pre-treatment for pars plana vitrectomy in proliferative diabetic retinopathy. Trial registration number: ChiCTR-ONC-16009520. Date of registration: October 20, 2016. URL of trial registry record: http://www.chictr.org.cn/searchprojen.aspx

## Background

Proliferative diabetic retinopathy (PDR) is the leading cause of blindness in the working-age population worldwide [1]. Conventional therapy for PDR combined with vitreous hemorrhage consists of vitrectomy plus retinal photocoagulation [2]. Stripping of the preretinal neovascularization membrane during surgery, however, especially in patients with advanced PDR, can induce bleeding or oozing of blood, seriously impairing the surgical field. Repeated bleeding prolongs the time of surgery and increases the frequency of intraoperative import and export of surgical instruments into the vitreous cavity, greatly increasing the likelihood of potential complications.

Vascular endothelial growth factor (VEGF) has been found to play an important role in many retinal vascular diseases [3–5]. Ranibizumab is a recombinant, monoclonal antibody fragment that inhibits VEGF [6], and intravitreal injection of ranibizumab (IVR) before vitrectomy has been reported to be an effective adjunct treatment for PDR [7]. Ranibizumab causes transient vasoconstriction, which may clinically resemble vascular regression. This "regression" of retinal neovascularization can occur in PDR, although clinical observations suggest that responses to anti-VEGF therapy are spontaneously reversed [8, 9].

Surgical trauma may be reduced by planning pars plana vitrectomy during this window period. Although anti-VEGF therapy can temporarily reduce leakage from diabetic neovascular lesions, it may be associated with tractional retinal detachment (TRD), a serious complication with poor prognosis in patients with PDR. Because anti-VEGF agents can induce fibrous proliferation, TRD can occur after their injection. Proliferative membrane formation without obvious TRD has been reported in one eye 9 days after intravitreal injection of conbercept [10]. Another study reported that the mean time from intravitreal injection of bevacizumab to TRD was 13 days (range, 3–31 days) and that anti-VEGF treatment can cause TRD for up to 3 days [11]. Intravitreal injection of conbercept 3–7 days before vitrectomy was found to increase the risk of TRD [12–14]. Although the effects of bevacizumab 24 h after injection have been reported [8], no study to date has evaluated the efficacy and safety of IVR 1–3 days before vitrectomy for the treatment of PDR with vitreous hemorrhage.

TRD is closely related to fibrous proliferation after anti-VEGF treatment. Retinal fibrosis in patients with PDR correlated significantly with the concentrations of connective tissue growth factor (CTGF) [15–17]. Increasing CTGF levels and/or decreasing VEGF levels can alter the balance between CTGF and VEGF, leading to a tilt in the angiofibrotic switch towards fibrosis [18–20].

The present study evaluated alterations in aqueous VEGF and CTGF concentrations before and after Ranibizumab injection and the correlations between these concentrations and clinical improvements. These findings may help determine a period during which IVR can be safely administered prior to surgery without worsening or inducing TRD.

## Methods

### Patients

The study was approved by the Ethical Review Board of the Second Xiangya Hospital of Central South University and conformed to the tenets of the Declaration of Helsinki. Participants were informed of the off-label use of IVR and provided a detailed description of the treatment. All participants provided written informed consent for this treatment.

In this prospective pilot study, participants were recruited prospectively in the Department of Ophthalmology of the Second Xiangya Hospital from November 2016 to July 2018. All subjects were Han Chinese. One eye of each subject was included. All subjects underwent a comprehensive ophthalmological evaluation, including visual examination, slit-lamp biological microscopy, measurement of intraoperative pressure (IOP) by non-contact tonometry, and fundus examination after pupil dilation with tropicamide (1%) and phenylephrine hydrochloride (2.5%). Patients were also examined by indirect ophthalmoscopy, ultrasonic biological microscopy, B-ultrasound scan, and optical coherence tomography (OCT). Patients were included if they had vitreous hemorrhage resulting from active PDR.

Patients were excluded if they had (1) neovascular glaucoma resulting from active PDR plus cataract or PDR combined with rhegmatogenous retinal detachment (RRD). If preoperative RRD was obscured by dense vitreous hemorrhage in patients with PDR, these patients were excluded if RRD was detected during surgery. Patients were also excluded if they had (2) a history of previous laser treatment or vitrectomy in the study eye; (3) a history of thromboembolic events, including myocardial infarction or cerebrovascular accidents; (4) a systemic inflammatory, autoimmune, or immunosuppressive disease; (5) a pre-existing ocular disease (retinal vein occlusion, retinal artery occlusion, or age-related macular degeneration); (6) uncontrolled hypertension, as defined by the guidelines of the seventh report of the joint National Committee on Prevention, Detection, Evaluation, and Treatment of High Blood Pressure; (7) a history of previous ocular surgery; or (8) a coagulation abnormality or current use of an anticoagulative medication other than aspirin.

The patients were randomly divided into three groups using a computer‐generated list of random numbers was used. Patients in the control group (group A) were administered a sham subconjunctival injection 3 days before vitrectomy, whereas patients in the 1-day IVR group (group B) and 3-day IVR group (group C) were administered IVR (0.5 mg/0.05 ml) 1 and 3 days before surgery, respectively.

Preliminary experimental results indicated that, to detect a reduction of VEGF between before and 1 or 3 days after IVR, with a one-sided 5% significance level and a power of 90%, a sample size of 16 patients per group was necessary.

Patients underwent fundus examinations and B-scanning before IVR and before vitrectomy to detect vitreous hemorrhage density and TRD. Vitreous hemorrhage density was graded as described [21], with Grade 0 indicating that no blood was present in the vitreous, and the entire retina was visible; Grade 1 as hemorrhage obscuring 1 to 5 clock hours of the retina; Grade 2 as hemorrhage obscuring 5 to 10 clock hours of the central and/or peripheral retina, or a large hemorrhage located posterior to the equator with varying clock hours of the anterior retina visible; Grade 3 as the presence of a red reflex with no retinal detail visible posterior to the equator; and Grade 4 as dense vitreous hemorrhage with no red reflex present. The effect of Ranibizumab on the extent of TRD prior to surgery was assessed by examining the study eye prior to IVR with ultrasonography (Acuson Sequoia 512 scanner, 14 MHz linear probe; Siemens Medical Solutions USA, Mountain View, CA, USA), and on the day of surgery by slit-lamp biomicroscopy and ultrasonography.

The total operation time, defined as the time from the first incision to the final operative closure, was measured intraoperatively. Immediately after surgery, the surgeon (J.Z.), who was masked to treatment allocation, completed a standardized questionnaire on the intraoperative outcomes, including the presence or absence of intraoperative bleeding, iatrogenic tears, relaxing retinotomy, use of endothermic, and use of silicone oil at the end of surgery. All surgical procedures were performed by a single surgeon (J.Z.) to avoid differences in technique and in data collection.

### Sample collection

Samples of aqueous humor and venous blood were collected during IVR (before IVR) and during vitrectomy (after IVR). Aqueous humor samples were collected through anterior chamber puncture using a 30-gauge needle and immediately frozen at -80℃ in the dark. Venous blood samples (5 ml) were collected in the fasting state and centrifuged at 1000 × g for 15 min, and the resulting plasma samples were divided into aliquots and stored at -80℃. To avoid damage to the blood-water barrier caused by surgical trauma, all samples were obtained at the beginning of the operation, prior to any conjunctival or intraocular procedure.

### Enzyme-linked immunosorbent assay (ELISA)

VEGF and CTGF concentrations were measured by VEGF (MultiSciences, Hangzhou, China) and CTGF (FibroGen, South San Francisco, CA) ELISA kits, according to the manufacturers’ protocols, as described [20–22]. Briefly, 50 µl/well aliquots of aqueous humor were pre-plated with monoclonal antibody on 96-well plates, and the plates were incubated at room temperature for 3 h. After washing three times, a second antibody was added to each well, and the plates were incubated at 37 ℃ for 3 h. Substrate was added to each well; the plates were incubated in the dark for 30 min at room temperature; and 100 µl stop solution (Multiskan Ascent; Thermo Fisher Scientific GmbH, Schwerte, Germany) were added to each well to terminate the reaction. All samples were prepared and measured on the same day using the same standard preparation methods. Each experiment was performed three times and their results were averaged.

### Data analysis

All data are expressed as the mean ± SD. The Shapiro–Wilk normality test was used to assess normality. Categorical covariates were assessed individually using χ^2^ tests. Intragroup differences in VEGF and CTGF concentrations and CTGF/log_10_ (VEGF) ratios were analyzed using Wilcoxon signed rank tests of two independent samples and paired-sample t tests. Correlations between VEGF and CTGF concentrations in aqueous humor in the PDR patients were analyzed by Spearman’s rank–order correlation tests. Best corrected visual acuity (BCVA) was converted to logMAR for statistical evaluation, and logMAR BCVAs before and 1 and 6 months after IVR were compared by repeated measure ANOVA. All statistical analyses were performed using SPSS 22.0 software, with p-values less than 0.05 considered statistically significant.

## Results

### Patients’ demographic data

This study included 48 eyes with PDR; of these, 16 eyes received subconjunctival injections of 0.05 ml BSS 3 days before vitrectomy, and 16 eyes each received IVR 1 and 3 days before vitrectomy. The mean ± SD ages of patients in these three groups were 53.1 ± 6.3, 46.9 ± 11.7, and 49.8 ± 10.1 years, respectively. Age, gender, incidence of hypertension, vitreous hemorrhage density, and TRD did not differ significantly in these three groups (all *p* > 0.05) (Table 1).

**Table 1:** Baseline demographic and clinical characteristics of the study population

Variables	Group A	Group B	Group C	*P*- value
Numbers	16	16	16	-
Age, y	53.1 ± 6.3	46.9 ± 11.7	49.8 ± 10.1	0.288*
Sex, male/female	8/8	9/7	10/6	0.776†
Hypertension (%)	43.75%	50%	31.25%	0.549†
Vitreous hemorrhage grading scale				0.557†
2	3	2	2	
3	4	3	2	
4	9	11	12	

^*^ Kruskal–Wallis H test. † χ^2^ test

### Functional and anatomical results

None of the patients receiving IVR 1 and 3 days before vitrectomy experienced a new occurrence of TRD after IVR treatment. The mean height of TRD after IVR increased slightly in patients who received IVR 3 days before vitrectomy (Table 2). At baseline, the mean BCVAs in patients who received BSS and IVR 1 and 3 days before vitrectomy were 2.006 ± 0.427 logMAR, 1.988 ± 0.463 logMAR, and 2.05 ± 0.412 logMAR, respectively, with no statistical significance. Three months after vitrectomy, the mean postoperative BCVAs in these three groups increased to 0.706 ± 0.277 logMAR, 0.488 ± 0.189 logMAR, and 0.463 ± 0.159 logMAR, respectively, with all of these values differing significantly from baseline (all *p* < 0.05) and VA improvement being significantly greater in the treated groups than in the sham group (*p* = 0.004) (Table 2). At the end of the operation, the retina was completely attached and vitreous hemorrhage was cleared in all eyes.

**Table 2:** Changes in LogMAR BCVA in the three groups of patients

Variables	Group A	Group B	Group C	F	*P*- value
LogMAR BCVA					
Baseline	2.006 ± 0.427	1.988 ± 0.463	2.05 ± 0.412	0.087	0.917
Month 3	0.706 ± 0.277	0.488 ± 0.189	0.463 ± 0.159	6.272	0.004

### Intraoperative findings

Table 3 shows the incidences of TRD before surgery, intraoperative bleeding, iatrogenic retinal breaks, use of endodiathermy, and use of silicone oil, and mean total surgical time in the three groups. Patients who received IVR 1 and 3 days before surgery showed significant differences in incidences of intraoperative bleeding, use of endodiathermy, and use of silicone oil tamponade and in mean total surgical time, from sham injected patients. However, there were no statistically significant differences in patient or surgical characteristics between the two IVR treated groups (*p* > 0.05) (Table 4).

**Table 3:** Tractional retinal detachment characteristics following injection of Ranibizumab 1 and 3 days prior to diabetic vitrectomy

Time point	Group A		Group B		Group C	
	Baseline	3 days	Baseline	1 days	Baseline	3 days
TRD characteristics from ultrasound						
Number of eyes with TRD	13(81.25%)	13(81.25%)	12(75%)	12(75%)	11(68.75%)	11(68.75%)
Number of eyes with macula TRD	9(56.25%)	9(56.25%)	7(43.75%)	7(43.75%)	7(43.75%)	7(43.75%)
Dimensions of TRD (mean (SD))						
Height (mm)	2.1 (1.7)	2.2 (1.7)	1.9 (1.7)	1.8 (1.7)	2.1 (1.6)	2.3 (1.9)
Base (mm)	6.7(5.4) × 6.8(5.1)	7.0(5.9) × 6.8(5.1)	7.4(5.3) × 6.3(4.7)	7.4(5.5) × 5.7(4.1)	8.2(5.0) × 8.5(5.3)	8.4(5.2) × 8.7(5.3)
Intraoperative TRD						
Absent	3		2		3	
Hammock	4		4		2	
Diffuse central	3		3		3	
Tabletop	3		3		3

**Table 4:** Intraoperative findings

Variables	Group A	Group B	Group C	Group A vs B	Group A vs C	Group B vs C
Intraoperative bleeding	12(75%)	2(12.5%)	1(6.25%)	*P* = 0.001	*P* = 0.000	*P* = 1
Endodiathermy	9(56.25%)	1(6.25%)	1(6.25%)	*P* = 0.006	*P* = 0.006	*P* = 1
Iatrogenic break	5(31.25%)	1(6.25%)	0(0%)	*P* = 0.172	*P* = 0.043	*P* = 1
Relaxing retinotomy	1(6.25%)	0(0%)	0(0%)	*P* = 1	*P* = 1	*P* = 1
Silicone-oil tamponade	9(56.25%)	2(12.5%)	2(12.5%)	*P* = 0.023	*P* = 0.023	*P* = 1
Balanced salt solution (BSS)	5(31.25%)	11(68.75%)	10(62.5%)			
Sterile air	2(12.5%)	3(18.75%)	4(25%)			
Surgical mean time (min)	110.625 ± 21.975	96.563 ± 16.705	86.25 ± 18.841	*P* = 0.05	*P* = 0.002	*P* = 0.112

### VEGF concentrations in aqueous humor and plasma before and after IVR

Prior to IVR, VEGF concentrations in aqueous humor in patients who received IVR 1 and 3 days before surgery were 467.958 ± 144.396 pg/ml (95% confidence interval [CI] 391.015–544.901 pg/ml) and 476.555 ± 277.061 pg/ml (95% CI 328.920–624.191 pg/ml), respectively. Following IVR, VEGF concentrations in aqueous humor of these groups were 85.186 ± 60.043 pg/ml (95% CI 53.191–117.181 pg/ml) and 108.398 ± 85.877 pg/ml (95% CI 62.638–154.159 pg/ml), respectively, showing that VEGF concentrations in aqueous humor decreased significantly after IVR in both groups (*p* < 0.05 each).

**Fig. 1:**
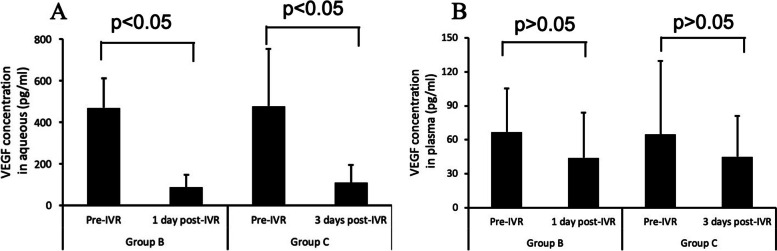
VEGF levels before and after IVR in aqueous humor (**A**) and plasma (**B**) samples. VEGF concentration in aqueous humor was significantly lower after than before IVR in the two groups of patients (*P* < 0.05 each)

Plasma VEGF concentrations in patients who received IVR 1 and 3 days before surgery were 66.831 ± 38.344 pg/ml (95% CI 46.399–87.263 pg/ml) and 64.599 ± 64.965 pg/ml (95% CI 29.981–99.216 pg/ml), respectively, before IVR and 43.999 ± 40.230 pg/ml (95% CI 22.569–65.440 pg/ml) and 45.086 ± 36.068 pg/ml (95% CI 25.866–64.305 pg/ml), respectively, after IVR, indicating that plasma VEGF concentrations in both groups did not differ significantly before and after IVR (*p* > 0.05 each) (Fig. 1).

### CTGF concentrations in aqueous humor and plasma before and after IVR

CTGF concentrations in patients who received IVR 1 and 3 days before surgery were 1053.477 ± 362.108 pg/ml (95% CI 860.523–1246.431 pg/ml) and 1394.441 ± 721.088 pg/ml (95% CI 1010.2–1778.682 pg/ml), respectively, before IVR and 1129.627 ± 382.730 pg/ml (95% CI 925.685–1333.57 pg/ml) and 1512.589 ± 669.342 pg/ml (95% CI 1155.921– 1869.256 pg/ml), respectively, after IVR, indicating that aqueous humor CTGF concentrations did not differ significantly before and after IVR in both groups.

**Fig. 2:**
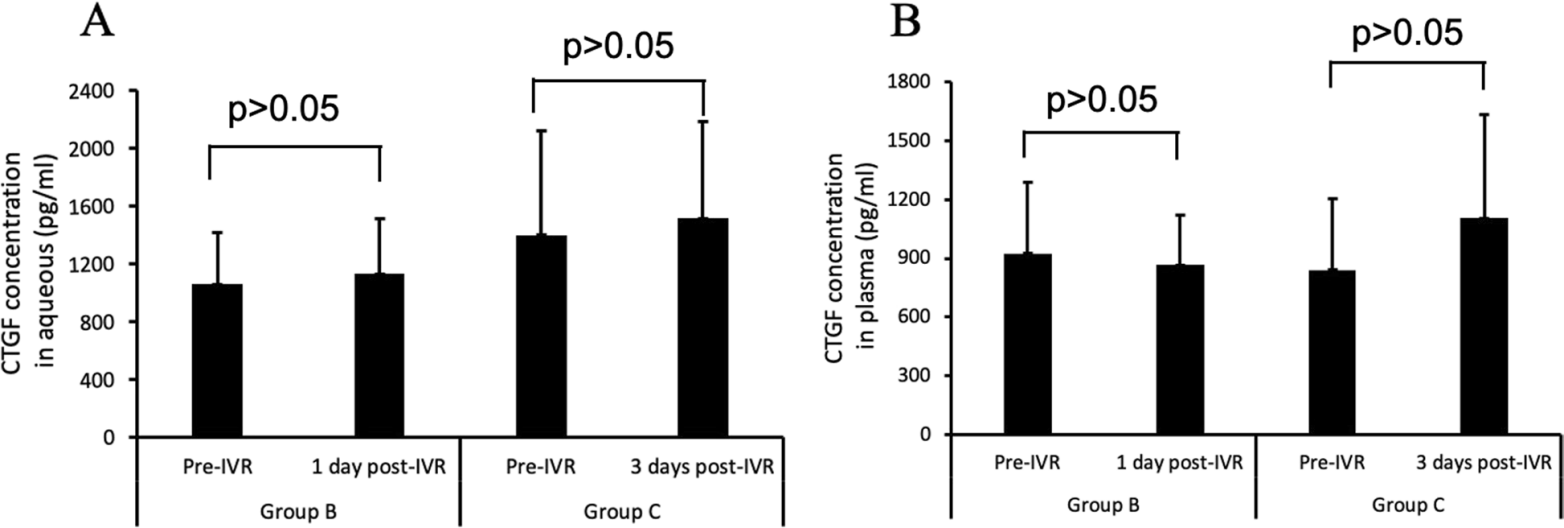
CTGF levels before and after IVR in aqueous humor (**A**) and plasma (**B**) samples. CTGF concentrations were unaffected by IVR in the two groups of patients (*P* > 0.05 each)

Plasma concentrations of CTGF in patients who received IVR 1 and 3 days before surgery were 925.999 ± 365.131 pg/ml (95% CI 731.434–1120.564 pg/ml) and 842.365 ± 361.432 pg/ml (95% CI 649.772–1034.958 pg/ml), respectively, before IVR and 865.99 ± 258.24 pg/ml (95% CI 728.385–1003.598 pg/ml) and 1106.744 ± 527.783 pg/ml (95% CI 825.508–1387.98 pg/ml), respectively, after IVR, indicating that plasma CTGF concentrations did not differ significantly before and after IVR in the two groups (all *p* > 0.05) (Fig. 2).

### CTGF/log_10_ (VEGF) ratio

Paired-sample t tests showed that the CTGF/log_10_ (VEGF) ratio before and after IVR did not differ significantly in patients who received IVR 1 day before vitrectomy (*P* = 0.051). This ratio, however, was significantly higher after than before IVR in patients who received IVR 3 days before vitrectomy (*P* = 0.046) (Table 5).

**Table 5:** CTGF/log_10_ (VEGF) ratios

Variables	Value	*P* value
Group B		
Ratio CTGF/log_10_(VEGF) before IVR	0.399 ± 0.141	0.051
Ratio CTGF/log_10_(VEGF) after IVR	0.724 ± 0.624	
Group C		
Ratio CTGF/log_10_(VEGF) before IVR	0.553 ± 0.284	0.046
Ratio CTGF/log_10_(VEGF) after IVR	0.803 ± 0.387	

### Correlation between the aqueous humor VEGF and CTGF in patients with PDR

**Fig. 3:**
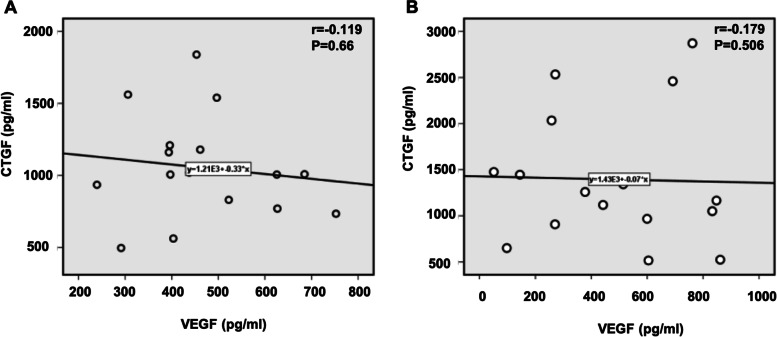
Correlation between the aqueous humor VEGF and CTGF concentrations in patients who received IVR 1 day (**A**) and 3 days (**B**) before vitrectomy

Spearman’s rank–order correlation analysis found no correlations between the concentrations of VEGF and CTGF in aqueous humor of patients who received IVR 1 day (r = -0.119; *P* = 0.66) and 3 days (r = -0.179; *P* = 0.506) before vitrectomy (Fig. 3).

### Correlations between the aqueous humor and plasma VEGF and CTGF levels in patients with PDR

No significant correlations were observed between aqueous humor and plasma VEGF concentrations of patients who received IVR 1 day (r = 0.174, *P* = 0.52) and 3 days (r = –0.218, *P* = 0.418) before surgery. Similarly, the concentrations of CTGF in aqueous humor and plasma of patients who received IVR 1 day (r = 0.049, *P* = 0.858) and 3 days (r = –0.156, *P* = 0.564) before surgery did not differ significantly (Table 6).

**Table 6:** Correlations of aqueous humor and plasma VEGF and CTGF concentrations in PDR patients with vitreous hemorrhage

Correlation	r	*P* value
Group B		
Aqueous humor VEGF – plasma VEGF	0.174	0.52
Aqueous humor CTGF – plasma CTGF	0.049	0.858
Group C		
Aqueous humor VEGF – plasma VEGF	-0.218	0.418
Aqueous humor CTGF – plasma CTGF	-0.156	0.564

## Discussion

To our knowledge, this study is the first to measure the effects of IVR on VEGF and CTGF concentrations in aqueous humor and serum and the correlations between these factors and clinical improvements in patients with PDR. Because the concentrations of VEGF are strongly correlated in the aqueous and vitreous humor of patients with PDR [23], aqueous samples were collected to measure the concentrations of VEGF and CTGF. We found that the concentration of VEGF in aqueous humor decreased significantly 1 day after IVR in patients with PDR, consistent with results showing that bevacizumab had a similar effect at 24 h [8]. This finding provides a theoretical basis for early traction dissection after injection. This study also found that the concentrations of VEGF in aqueous humor were significantly lower 3 days after than before IVR. Up-regulation of VEGF can promote angiogenesis and increase vascular permeability [22, 24, 25]. VEGF plays a key role in the pathogenesis of diabetic retinopathy as a mediator between neovasculogenesis and permeability. VEGF levels are significantly higher in patients with than without PDR, with VEGF level being directly proportional to the growth of new blood vessels and leakage [26, 27] and the severity of diabetic retinopathy [28]. The present study found that, compared with eyes that underwent vitrectomy in the absence of IVR, those who received IVR experienced intraoperative improvements. Specifically, the rates of intraoperative bleeding, use of intraocular electric coagulation, and need for silicone oil tamponade, as well as operation time, were significantly lower in patients who received IVR 1 and 3 days before vitrectomy compared with the sham treated group. Injection of Ranibizumab reduced bleeding during the cutting phase and during the removal of fibrous vascular tissue, facilitated membrane dissection, and made surgery easier and safer. However, the rates of intraoperative bleeding, use of intraocular electric coagulation, and need for silicone oil tamponade, as well as operation time, did not differ significantly in patients who received IVR 1 and 3 days before vitrectomy.

In agreement with results showing that bevacizumab was effective 24 h after administration [8], the present study confirmed that IVR 1 day before vitrectomy was effective for patients with PDR, as it significantly improved intraoperative conditions.

The concentrations of CTGF in aqueous humor 1 and 3 days after IVR did not differ significantly from the concentration before IVR. These time periods may have been too soon after IVR to detect significant changes in CTGF in aqueous humor. In addition, because VEGF can up-regulate CTGF, a decrease in VEGF may down-regulate CTGF, especially during early stages of VEGF inhibition [29, 30]. Moreover, because CTGF binds to the extracellular matrix in its natural state through the heparin binding domain, soluble CTGF that circulates freely in eye fluid may become degraded [31]. Finally, the number of eyes included in this study may have been too small to detect a significant difference in CTGF concentration.

CTGF is an indicator of intraocular fibrosis. Evidence has shown that CTGF plays an important role in promoting fibrosis and inducing wound healing in the body and eyes. Elevated levels of CTGF have been associated with proliferative vitreoretinopathy, choroid neovascularization, and PDR. In addition, the ratio of CTGF to VEGF concentration is an important predictor of vascular fibrosis transformation in PDR. Increasing CTGF and/or reducing VEGF concentrations can alter the balance between CTGF and VEGF, leading to a vascular fibrosis switch that causes fibrosis. Anti-VEGF therapy can lead to the development of TRD [18]. We did not observe significant TRD progression 1 and 3 days after IVR, suggesting that an interval of 1 to 3 days between IVR and vitrectomy is a safe window to reduce intraoperative hemorrhage during membrane dissection, facilitating surgery without worsening pre-existing TRD or inducing new TRD. IVR 1–3 days before vitrectomy can also reduce hospital length of stay and patient economic burden when compared with IVR 3–7 days before PPV.

Because the ratio of CTGF to VEGF concentrations was found to be the strongest predictor of the degree of fibrosis [17, 32], CTGF/log_10_ (VEGF) ratios were compared before and after IVR. We found CTGF/log_10_ (VEGF) ratio was significantly increased 3 days after IVR, mainly due to a reduction in VEGF levels. This change may be related to intraocular fibrosis activity, suggesting that after IVR, the risk of fibrosis occurrence or progression increases over time, suggesting that vitrectomy soon after IVR would benefit patients.

The present study found no correlation between CTGF and VEGF concentrations in aqueous humor. In contrast, a previous study found significant correlations between CTGF and log_10_ (VEGF) concentrations in the vitreous humor of patients with PDR [16]. Additional studies are required to clarify this discrepancy.

This study took some steps to decrease the potential inaccuracies. Plasma concentrations of VEGF and CTGF were measured before and after IVR, but did not differ significantly. In addition, correlation analysis showed that no association between plasma and aqueous levels of VEGF and CTGF in either IVR group. These results suggest that the elevated levels of VEGF were not associated with systemic diseases but are produced locally by increased retinal secretion, followed by leakage into the anterior chamber.

This study had several limitations, including the small number of included subjects. In addition, despite measuring the levels of CTGF and VEGF in aqueous humor, we could not establish a cause-and-effect relationship between them. In addition, although we found that IVR improved the intraoperative conditions of these patients, we did not conduct a postoperative analysis. Additional studies are needed to determine long-term outcomes of IVR combined with vitrectomy in patients with PDR.

## Conclusions

This pilot study showed that preoperative IVR is an effective and safe strategy for the surgical treatment of severe PDR combined with vitreous hemorrhage. IVR administered 1 and 3 days before surgery can significantly reduce VEGF concentrations in aqueous humor and effectively improve intraoperative conditions without causing TRD. Prospective randomized studies with larger sample sizes are needed to further investigate the effect of IVR 1 or 3 days before vitrectomy for PDR.

## Data Availability

The datasets used and/or analyzed during the current study are available from the corresponding author on reasonable request.

## Abbreviations

VEGF: Vascular endothelial growth factor
IVR: Intravitreal injection of Ranibizumab
PDR: Proliferative diabetic retinopathy
CTGF: Connective tissue growth factor
TRD: Tractional retinal detachment
ELISA: Enzyme-linked immunosorbent assay
BCVA: Best corrected visual acuity
